# Post-operative acute kidney injury and five-year risk of death, myocardial infarction, and stroke among elective cardiac surgical patients: a cohort study

**DOI:** 10.1186/cc13158

**Published:** 2013-12-12

**Authors:** Malene Kærslund Hansen, Henrik Gammelager, Martin Majlund Mikkelsen, Vibeke Elisabeth Hjortdal, J Bradley Layton, Søren Paaske Johnsen, Christian Fynbo Christiansen

**Affiliations:** 1 Department of Clinical Epidemiology, Aarhus University Hospital, Oluf Palmes Allé 43-45, 8200 Aarhus N, Denmark; 2 Department of Dermato- and Venerolgy, Aarhus University Hospital, P.P. Ørums Gade 11, 8000 Aarhus C, Denmark; 3 Department of Cardiothoracic and Vascular Surgery, Aarhus University Hospital, Brendstrupgårdsvej 100, 8200 Aarhus N, Denmark; 4 Department of Epidemiology, University of North Carolina at Chapel Hill, 2106 McGavran-Greenberg Hall CB 7435, Chapel Hill, NC 27599-7435, USA

## Abstract

**Introduction:**

The prognostic impact of acute kidney injury (AKI) on long-term clinical outcomes remains controversial. We examined the five-year risk of death, myocardial infarction, and stroke after elective cardiac surgery complicated by AKI.

**Methods:**

We conducted a cohort study among adult elective cardiac surgical patients without severe chronic kidney disease and/or previous heart or renal transplant surgery using data from population-based registries. AKI was defined by the Acute Kidney Injury Network (AKIN) criteria as a 50% increase in serum creatinine from baseline level, acute creatinine rise of ≥26.5 μmol/L (0.3 mg/dL) within 48 hours, and/or initiation of renal replacement therapy within five days after surgery. We followed patients from the fifth post-operative day until myocardial infarction, stroke or death within five years. Five-year risk was computed by the cumulative incidence method and compared with hazards ratios (HR) from a Cox proportional hazards regression model adjusting for propensity score.

**Results:**

A total of 287 (27.9%) of 1,030 patients developed AKI. Five-year risk of death was 26.5% (95% CI: 21.2 to 32.0) among patients with AKI and 12.1% (95% CI: 10.0 to 14.7) among patients without AKI. The corresponding adjusted HR of death was 1.6 (95% CI: 1.1 to 2.2). Five-year risk of myocardial infarction was 5.0% (95% CI: 2.9 to 8.1) among patients with AKI and 3.3% (95% CI: 2.1 to 4.8) among patients without AKI. Five-year risk of stroke was 5.0% (95% CI: 2.8 to 7.9) among patients with AKI and 4.2% (95% CI: 2.9 to 5.8) among patients without AKI. Adjusted HRs were 1.5 (95% CI: 0.7 to 3.2) of myocardial infarction and 0.9 (95% CI: 0.5 to 1.8) of stroke.

**Conclusions:**

AKI, within five days after elective cardiac surgery, was associated with increased five-year mortality and a statistically insignificant increased risk of myocardial infarction. No association was seen with the risk of stroke.

## Introduction

Acute kidney injury (AKI) occurs in up to 30% of patients undergoing cardiac surgery and has been reported to be associated with increased mortality [1,2]. AKI is defined as an abrupt decline of kidney function with severity ranging from mild kidney dysfunction to complete renal failure with the need for acute dialysis. Recent classification systems divide AKI into three severity levels based on changes in serum creatinine level and/or urine output [3].

Previous studies in cardiac surgical patients have mainly focused on severe AKI requiring dialysis [4]. Lately, focus has shifted towards the mortality impact of less severe AKI as defined by either Risk, Injury, Failure, Loss of function, and End-stage renal disease (RIFLE) or Acute Kidney Injury Network (AKIN) criteria. Only three studies have examined the long-term prognosis (that is, beyond 90 days) of cardiac surgery complicated by less severe AKI [5-7]. These studies have found that AKI is associated with a 40 to 50% increase in long-term mortality and that AKI is associated with a higher one-year risk of major adverse cardiac events compared to patients without AKI. The studies were limited by baseline serum creatinine being estimated by the Modification of Diet in Renal Disease equation rather than measured, inclusion of both acute and elective surgical patients, and incomplete follow-up data [6,7].

More insight into the prognostic role of AKI in elective cardiac surgical patients is needed, as AKI occurs frequently and may have devastating consequences for the patient. Increased awareness of AKI could, therefore, potentially facilitate a more prophylactic treatment strategy among high-risk patients.

We, therefore, conducted a cohort study of elective cardiac surgical patients with detailed pre-, peri- and post-operative information to examine the prognostic role of early AKI on long-term risk of major adverse clinical outcomes including death, myocardial infarction and stroke.

## Materials and methods

### Design and setting

We conducted the study at the Department of Cardiothoracic and Vascular Surgery, Aarhus University Hospital, Denmark. The hospital provides cardiac surgery for a mixed rural-urban population of approximately 1.2 million inhabitants (20% of the total Danish population) in the Central Denmark Region. The Danish National Health Service provides tax-funded medical care for all Danish residents. Due to the unique Central Personal Registry number assigned to each Danish citizen at birth and to residents on immigration, it is possible to make accurate linkage to patient and registries at an individual level [8]. The study was approved by the Danish Data Protection Agency and the Regional Ethics Committee (record number: 2013-41-1516).

### Elective cardiac surgical patients

During the period from 1 April 2005 to 8 October 2007, a total of 2,215 patients underwent acute and elective cardiac surgery at the Department of Cardiothoracic and Vascular Surgery, Aarhus University Hospital, Denmark. Patient screening and recruitment was done with the assistance of a project nurse working half-time, thus approximately 50% of the total population could be screened consecutively. Patients were included in the study database on the basis of: 1) age (≥18 years old); and 2) elective cardiac surgery (surgery performed more than two days after planning the procedure) including valve surgery, on- and off-pump coronary artery bypass grafting, thoracic aortic surgery, pulmonary thromboendarterectomy, ventricular aneurysm, adult congenital heart disease procedures, or combined procedures. Exclusion criteria were: 1) severe pre-existing chronic kidney disease (serum creatinine >200 μmol/L (2.3 mg/dL)); and/or 2) previous heart or renal transplant surgery. The nurse prospectively collected information and completed a case-report-form for each patient included in the study containing baseline characteristics such as: smoking; body mass index (BMI); diabetes mellitus; dyslipidemia; blood pressure, and in-hospital peri-operative information. Every patient included in the study gave their informed consent to participate.

### Acute kidney injury

We used the regional laboratory database to obtain pre- and post-operative laboratory measurements. This population-based database contains information on all patient tests analyzed since 1997, including analyses codes, measurement units, dates of test collection and results [9]. Measurements of plasma creatinine - equivalent to serum creatinine - were used to classify patients as either AKI or non-AKI according to the serum creatinine criteria in the AKIN classification [3,10]. We did not include the urine output criteria. The term AKI included all AKIN stages, and was further subdivided according to the individual AKIN stages. For each patient, a pre-operative blood sample was collected 10 days prior to surgery. Accordingly, the baseline creatinine was available for all study participants. The peak post-operative measurement of creatinine from surgery start until day five was compared to baseline creatinine to assign AKI status.

### Study endpoints

Information on all-cause death was obtained through linkage to the Danish Civil Registration System [8]. This system includes information on all changes in vital status, migration and exact date of death for the Danish population since 1968 and is electronically updated daily.

Causes of death (both immediate and underlying) were studied through linkage to the Danish Registry of Causes of Death, which contains date and causes of death according to the International Classification of Diseases 10^th^ revision (ICD-10) classification (Additional file 1) [11].

Data regarding myocardial infarction and stroke (including both ischemic and hemorrhagic stroke) were obtained from the Danish National Registry of Patients (DNRP) (Additional file 1) [12]. The DNRP is a nationwide registry established in 1977 and includes civil registration number, hospital, department, discharge diagnosis, as well as surgical and diagnostic procedures. Since 1994, diagnoses have been coded using the ICD-10. We included all first-time hospitalizations with a discharge diagnosis of the specified outcome, occurring after index admission for surgery. Date of diagnosis was defined as the date of hospitalization (not including out-patient visits). Outcomes occurring during index admission for surgery, that is, from admission date until discharge date, were excluded for the concerned analysis.

### Covariates

Information on potential confounding factors was obtained from a pre-operative interview and medical records [13]. The included covariates were gender, age, smoking habits (present, never, previous), BMI, history of ischemic peripheral disease, previous stroke, previous myocardial infarction, history of arrhythmias, diabetes, dyslipidemia and hypertension. In addition, we obtained data on pre-existing comorbidity based on diagnoses from the DNRP (ICD-8 and ICD-10) since 1977 to compute the Charlson Comorbidity Index (CCI) scores. The CCI includes 19 disease categories with an assigned weight, and the sum of the weights defines the level of comorbidity. Patients were categorized as having low (score 0), medium (score 1 to 2) and high (score ≥3) levels of comorbidity (Additional file 2) [14]. The Western Denmark Heart Registry established in 1999 is a regional administrative and clinical register including detailed records on baseline patient characteristics and data regarding all cardiac procedures as well as corresponding covariates [15]. From this registry we obtained procedural characteristics including type of surgery, extra-corporal circulation and the EuroSCORE (European System for Cardiac Operative Risk Evaluation). The EuroSCORE assigns the patient an operative mortality risk based on patient-, cardiac- and operation-related factors [16].

### Statistical analyses

We followed patients from day five after surgery (that is, after assignment of AKI status) until death or emigration occurred or up to five years.

For the full cohort the cumulative incidence method was used to compute one- and five-year absolute risk of death, myocardial infarction and stroke. Death was considered a competing risk in the estimation of the risk of myocardial infarction and stroke. We computed five-year unadjusted and adjusted hazard ratios (HRs) for death, myocardial infarction and stroke using a Cox proportional hazards regression model. The assumption of proportional hazards was examined graphically and fulfilled for the whole time period and for every outcome.

We computed a propensity score, which predicted the probability of developing AKI conditional on the observed baseline covariates, using multivariable logistic regression. Modeling the exposure, rather than the outcome propensity scores, efficiently allows for simultaneous control for a large number of potentially confounding factors in studies such as ours where we have few outcomes but many exposed [17]. The included covariates were: gender, age, smoking, BMI, history of ischemic peripheral disease, previous stroke, previous myocardial infarction, history of arrhythmias, diabetes mellitus, dyslipidemia, hypertension, CCI, baseline creatinine, EuroSCORE, type of surgical procedure (valve, Coronary Artery Bypass Grafting (CABG), combined valve and CABG, others), and extra corporal circulation.

In the analyses of the full cohort, the HR was adjusted for the propensity score as a continuous variable. Furthermore, we performed a propensity score matched analyses which aimed to match each AKI patient with the non-AKI patient with the nearest propensity score within a maximum caliper range of ± 0.025 and without replacement. In this manner we were able to match 257 (89.5%) of 287 AKI patients with a non-AKI patient. Covariates were adequately balanced after propensity score matching, as evidenced by a standardized difference of each covariate to values below 0.1 [18]. In the matched cohort we also computed the cumulative risk and HRs for each outcome, stratified on the matched pairs.

We examined the causes of death for the full cohort, including both immediate and underlying causes of death. Hence, a patient may be registered with more than one cause of death. Causes were listed in disease categories and estimates given as percentages of the total number of causes of death according to AKI status.

Analyses were performed using the statistical software package Stata® 12.0 package (StataCorp LP, College Station, TX, USA).

## Results

**Figure 1:**
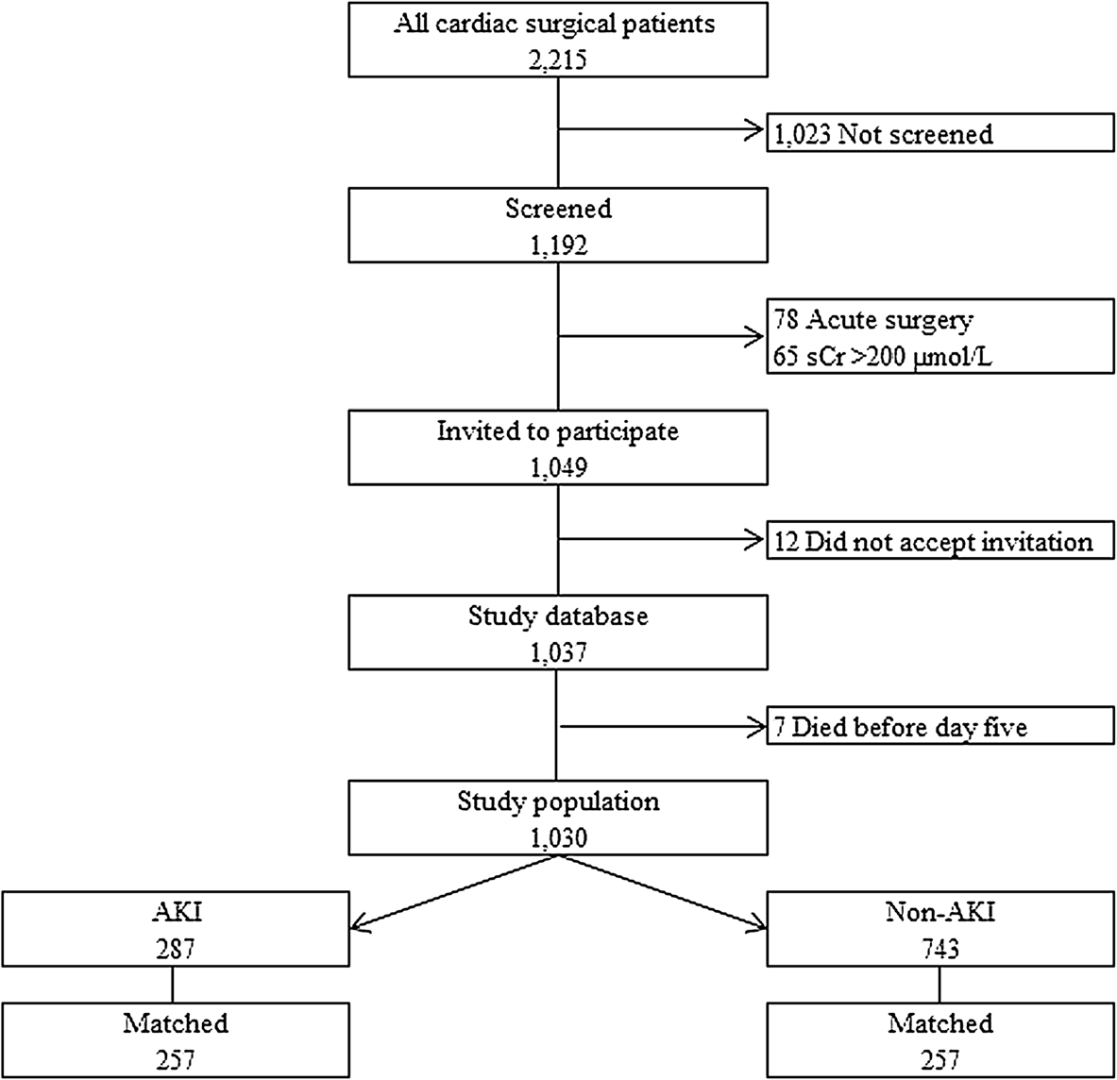
Flowchart.

The study population comprised 1,030 patients (Figure 1). A total of 27.9% (287 of 1,030) had an episode of AKI during the first five post-operative days; these included 82.9% (238 of 287) of patients in AKI stage 1 and 17.1% (49 of 287) of patients in AKI stage 2 or 3. AKI patients were older, more likely to have a history of stroke, arrhythmias and diabetes, had a higher comorbidity score and a higher EuroSCORE. Mean baseline creatinine was 94.2 μmol/L for AKI patients and 81.4 μmol/L for non-AKI patients (Table 1). In the matched cohort the covariates were equally distributed.

**Table 1:** Descriptive data of patients undergoing elective cardiac surgery in the full cohort and matched cohort

		**Full cohort**		**Matched cohort**	
	**Total**^**a**^	**AKI**^**a**^	**non-AKI**^**a**^	**AKI**^**a**^	**non-AKI**^**a**^
Clinical features	n = 1,030	n = 287	n = 743	n = 257	n = 257
**Pre-operative characteristics**					
Male gender	750 (72.8)	209 (72.8)	541 (72.8)	190 (73.9)	189 (73.5)
Age (years), mean (IQR)	65.8 (59 to 75)	70.0 (64 to 78)	64.1 (58 to 73)	69.4 (63 to 77)	69.3 (65 to 76)
Smoker					
Present	487 (47.3)	147 (51.2)	340 (45.8)	130 (50.6)	129 (50.2)
Never	357 (34.7)	88 (30.7)	269 (36.2)	82 (31.9)	83 (32.3)
Previous	186 (18.1)	52 (18.1)	134 (18.0)	45 (17.5)	45 (17.5)
BMI (kg/m^2^)					
<25	287 (27.9)	85 (29.6)	202 (27.2)	72 (28.0)	63 (24.5)
25 to 30	425 (41.3)	110 (38.3)	315 (42.4)	103 (40.0)	100 (38.9)
>30	318 (30.9)	92 (32.1)	226 (30.4)	82 (31.9)	94 (36.6)
Previous ischemic peripheral disease	57 (5.5)	19 (6.6)	38 (5.1)	17 (6.6)	13 (5.1)
Previous stroke	104 (10.1)	37 (12.9)	67 (9.0)	32 (12.5)	29 (11.3)
Previous myocardial infarction	256 (24.9)	66 (23.0)	190 (25.6)	63 (24.5)	65 (25.3)
History of arrhythmias	154 (15.0)	60 (20.9)	94 (12.7)	49 (19.1)	51 (19.8)
Diabetes mellitus	166 (16.1)	58 (20.2)	108 (14.5)	50 (19.5)	55 (21.4)
Dyslipidemia	570 (55.3)	156 (54.3)	414 (55.7)	141 (54.9)	147 (57.2)
Hypertension	585 (56.8)	166 (57.8)	419 (56.4)	146 (56.8)	153 (59.5)
Normal <140 and <90^b^	455 (44.2)	121 (42.2)	324 (43.6)	111 (43.2)	104 (40.5)
Grade I 140 to 159 or 90 to 99^b^	306 (29.7)	80 (27.9)	226 (31.4)	70 (27.2)	91 (23.7)
Grade II 160 to 179 or 100 to 109^b^	189 (18.4)	61 (21.3)	128 (17.2)	56 (21.8)	42 (16.3)
Grade III > =180 or > =110^b^	90 (8.7)	25 (8.7)	65 (8.8)	20 (7.8)	20 (7.8)
Charlson comorbidity index					
Low (score 0)	396 (38.5)	91 (31.7)	305 (41.5)	86 (33.5)	91 (35.0)
Medium (score 1 to 2)	456 (44.3)	135 (47.0)	321 (43.2)	119 (46.3)	112 (43.6)
High (score >3)	178 (17.3)	61 (21.3)	117 (15.7)	52 (20.2)	54 (21.0)
Baseline creatinine (μmol/L), mean (IQR)	85.0 (68 to 98)	94.2 (73 to 109)	81.4 (66 to 92)	90.9 (72 to 107)	91.3 (73 to 105)
euroSCORE, mean (IQR)^c^	5.2 (3 to 7)	6.4 (4 to 8)	4.7 (3 to 7)	6.1 (4 to 8)	6.1 (4 to 8)
Low risk (score 0 to 2)	199 (19.3)	30 (10.5)	169 (22.8)	30 (11.7)	28 (10.9)
Medium risk (score >2 to 5)	369 (35.8)	82 (28.6)	287 (38.6)	78 (30.4)	81 (31.5)
High risk (score >5)	462 (44.9)	175 (70.0)	287 (38.6)	149 (58.0)	148 (57.6)
**Surgical procedure characteristics**					
Type of surgery					
Valve^d^	313 (30.4)	84 (29.3)	229 (30.8)	77 (30.0)	78 (30.4)
CABG	372 (36.1)	85 (29.6)	287 (38.6)	80 (31.1)	71 (27.6)
Valve and CABG	158 (15.3)	59 (20.6)	99 (13.3)	51 (19.8)	54 (17.1)
Other^e^	187 (18.2)	59 (20.6)	128 (17.2)	49 (19.1)	54 (17.1)
Extra corporal circulation	910 (88.4)	255 (88.9)	655 (88.2)	227 (88.3)	223 (86.8)

^a^Values are expressed as counts (percentage) unless otherwise indicated.

^b^Measured in mmHg.

^c^EuroSCORE: A risk score for the operative mortality.

^d^Valve: Aorta, mitral, tricuspidal.

^e^Other: Pulmonary valve surgery, coarctatio, subvalvular membrane, ventricular aneurysme, ventricular septum defect, atrial septum defect, pulmonary thromboendatrerectomi, thoracic aorta.

AKI*,* Acute Kidney Injury; BMI*,* Body Mass Index; CABG*,* Coronary Artery Bypass Grafting; IQR*,* Interquartile Range.

**Figure 2:**
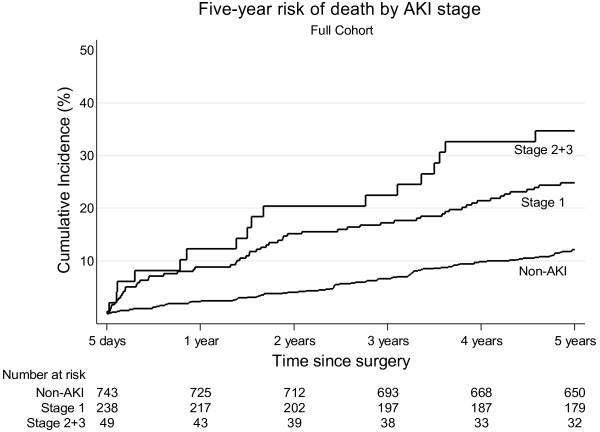
Kaplan Meier curve illustrating the five-year risk of death in the full cohort.

**Figure 3:**
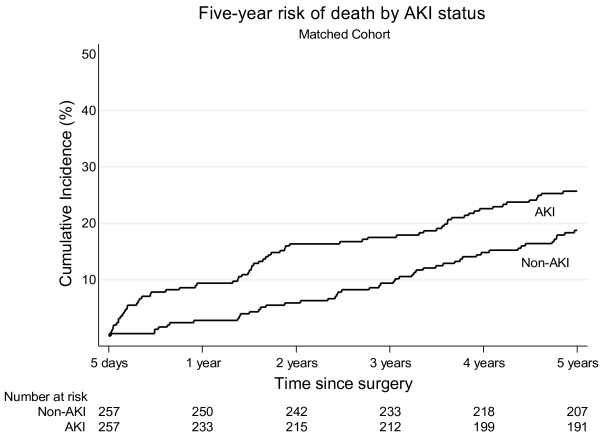
Kaplan Meier curve illustrating the five-year risk of death in the matched cohort.

Three patients emigrated during follow-up. Total follow-up time was 4,699 person-years with a median duration of five years. In the full cohort, a total of 166 patients died during the five years of follow-up (76 AKI patients and 90 non-AKI patients). We found a five-year cumulative risk of death of 26.5% (95% CI: 21.2 to 32.0) among AKI patients compared with 12.1% (95% CI: 10.0 to 14.7) among non-AKI patients. Adjusted HR was 1.6 (95% CI: 1.1 to 2.2). When stratifying according to AKI stage we found a progressively higher mortality with advancing AKI stage: Five-year cumulative risk of death in AKI stage 1 of 24.8% (95% CI: 19.5 to 31.2) and AKI stage 2 and 3 of 34.7% (95% CI: 23.2 to 49.7) (Figure 2). The adjusted HRs were 1.4 (95% CI: 1.0 to 2.1) for AKI stage 1 and 2.3 (95% CI: 1.4 to 3.9) for AKI stage 2 and 3 compared to non-AKI patients (Table 2). In the matched cohort, we found a five-year risk of death of 18.7% (95% CI: 14.5 to 24.1) for non-AKI patients and 25.7% (95% CI: 20.8 to 31.5) for AKI patients. The HR of death was 1.6 (95% CI: 1.1 to 2.4) comparing AKI with non-AKI patients (Table 3, Figure 3).

**Table 2:** Five-year results for death, myocardial infarction, and stroke by AKI status in the full cohort

	**Events**	**Number at period start**	**One-year risk**	**Five-year risk**	**Hazard ratio**	
					**Crude**	**Adjusted**^**a**^
Endpoint	n	n	% (95% CI)	% (95% CI)	(95% CI)	(95% CI)
**Death**						
Non-AKI	90	743	2.3 (1.4 to 3.7)	12.1 (10.0 to 14.7)	1 (reference)	1 (reference)
AKI	76	287	9.4 (6.6 to 13.4)	26.5 (21.2 to 32.0)	2.4 (1.8 to 3.3)	1.6 (1.1 to 2.2)
Stage 1	59	238	8.8 (5.8 to 13.2)	24.8 (19.8 to 30.8)	2.3 (1.6 to 3.1)	1.4 (1.0 to 2.1)
Stage 2 + 3	17	49	12.2 (5.7 to 25.2)	34.7 (23.2 to 49.7)	3.4 (2.0 to 5.7)	2.3 (1.4 to 3.9)
**Myocardial infarction**						
Non-AKI	23	708	1.4 (0.7 to 2.5)	3.3 (2.1 to 4.8)	1 (reference)	1 (reference)
AKI	14	278	1.8 (0.7 to 3.9)	5.0 (2.9 to 8.1)	1.7 (0.9 to 3.3)	1.5 (0.7 to 3.2)
Stage 1	11	230	1.7 (0.6 to 4.1)	4.7 (2.6 to 7.6)	1.6 (0.8 to 3.3)	1.4 (0.7 to 3.1)
Stage 2 + 3	3	48	2.1 (0.2 to 9.6)	6.3 (1.6 to 15.4)	2.2 (0.7 to 7.4)	2.0 (0.6 to 6.9)
**Stroke**						
Non-AKI	31	736	1.6 (0.9 to 2.8)	4.2 (2.9 to 5.8)	1 (reference)	1 (reference)
AKI	14	282	2.1 (0.9 to 4.4)	5.0 (2.8 to 7.9)	1.3 (0.7 to 2.4)	0.9 (0.5 to 1.8)
Stage 1	10	236	1.8 (0.7 to 3.9)	5.0 (2.8 to 7.9)	1.1 (0.5 to 2.2)	0.8 (0.4 to 1.6)
Stage 2 + 3	4	46	2.1 (0.9 to 4.4)	3.2 (1.6 to 5.7)	2.5 (0.9 to 7.0)	1.8 (0.6 to 5.3)

^a^Adjusted for propensity score.

AKI*,* Acute Kidney Injury; CI*,* Confidence Interval.

**Table 3:** Five-year results for death, myocardial infarction, and stroke by AKI status in the matched cohort

	**Events**	**Number at period start**	**One-year risk**	**Five-year risk**	**Hazard ratio**
Endpoint	n	n	% (95% CI)	% (95% CI)	(95% CI)
**Death**					
Non-AKI	48	257	2.7 (1.3 to 5.6)	18.7 (14.5 to 24.1)	1 (reference)
AKI	66	257	9.3 (6.4 to 13.6)	25.7 (20.8 to 31.5)	1.6 (1.1 to 2.4)
**Myocardial infarction**					
Non-AKI	7	242	0.4 (0.0 to 2.1)	2.9 (1.3 to 5.6)	1 (reference)
AKI	11	248	1.6 (0.5 to 3.8)	4.4 (2.4 to 7.5)	1.1 (0.4 to 3.2)
**Stroke**					
Non-AKI	17	252	3.2 (1.5 to 5.9)	6.7 (4.1 to 10.3)	1 (reference)
AKI	10	252	2.0 (0.8 to 4.3)	4.0 (2.0 to 6.9)	0.5 (0.2 to 1.2)

AKI*,* Acute Kidney Injury; CI*,* Confidence Interval.

When analyzing the risk of myocardial infarction, 35 non-AKI patients and 9 AKI-patients were excluded because they received their myocardial infarction diagnosis during the index admission for surgery. We found a five-year cumulative risk of myocardial infarction of 5.0% (95% CI: 2.9 to 8.1) among AKI patients and 3.3% (95% CI: 2.1 to 4.8) among non-AKI patients. The adjusted HR was 1.5 (95% CI: 0.7 to 3.2) (Table 2). In the matched cohort, we found similar five-year risks and a HR of 1.1 (95% CI: 0.4 to 3.2) (Table 3).

When analyzing the risk of stroke, seven non-AKI patients and five AKI-patients were excluded due to receiving their stroke diagnosis during the index admission for surgery. We found a five-year cumulative risk of stroke of 5.0% (95% CI: 2.8 to 7.9) among AKI patients and 4.2% (95% CI: 2.9 to 5.8) among non-AKI patients. The adjusted HR was 0.9 (95% CI: 0.5 to 1.8) for AKI patients compared with non-AKI patients (Table 2). In the matched cohort, we found similar five-year risks and a HR of 0.5 (95% CI: 0.2 to 1.2) (Table 3).

**Figure 4:**
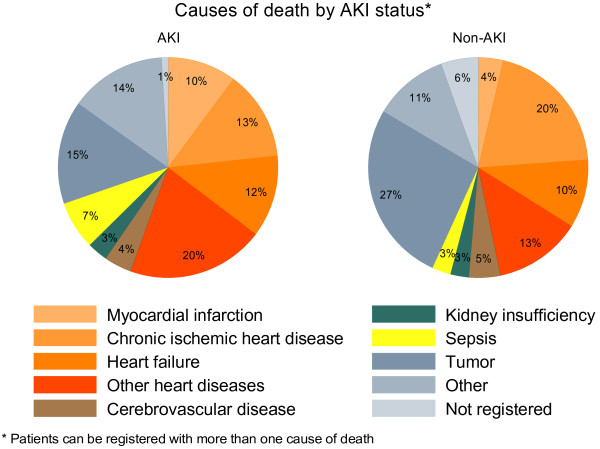
Causes of death by AKI status.*

Heart disease was registered as cause of death in 55% (95% CI: 45 to 66) of causes among AKI patients and 47% (95% CI: 37 to 57) of causes among non-AKI patients (Figure 4). Myocardial infarction was registered as the cause of death in 10% (95% CI: 5 to 18) of causes among AKI patients and 4% (95% CI: 1 to 9) of causes among non-AKI patients. Kidney insufficiencies and cerebrovascular diseases (including stroke) were equally distributed between the two groups. Data on cause of death were not available on seven patients.

We found that estimated Glomerular Filtration Rate (GFR) was slowly declining in the follow-up period after elective cardiac surgery (Additional file 3).

## Discussion

### Key results

We found that more than one out of four adult elective cardiac surgical patients without pre-existing severe kidney impairment developed AKI according to the AKIN criteria within five days after surgery. AKI was associated with increased mortality up to five years after elective cardiac surgery. Although statistically imprecise, AKI may be associated with an increased risk of myocardial infarction, but there was no association with the risk of stroke.

### Existing studies

Only three studies have examined the long-term impact of RIFLE/AKIN defined AKI following cardiac surgery [5-7]. In a US cohort of 2,973 acute and elective cardiothoracic surgical patients, a total of 1,265 patients (43%) experienced an episode of AKI during admission. They found a 10-year adjusted HR for death of 1.39 (95% CI: 1.23 to 1.57) [6]. They observed a higher proportion of patients who developed AKI compared to our findings (27.9%), which may partly be explained by the estimation of baseline creatinine by the Modification of Diet in Renal Disease equation rather than measuring creatinine. Studies have reported that the Modification of Diet in Renal Disease equation overestimates the incidence of AKI [19]. This misclassification may bias the association between AKI and death towards a lower risk of death among AKI patients. Furthermore, the inclusion of acute patients will tend towards a higher proportion of patients developing AKI. However, the HR estimate was in concordance with our findings (adjusted HR of 1.6 (95% CI: 1.1 to 2.2). Tsai *et al*. studied the long-term impact of RIFLE-defined AKI after surgery for aortic dissection. AKI occurred in 135 (52.7%) of 256 patients and they found an adjusted one-year HR for death of 2.6 (95% CI: 1.0 to 6.3) [7]. Finally, Gallagher *et al*. found in a propensity score matched cohort an adjusted five-year HR for death of 1.52 (95% CI: 1.19 to 1.93) after CABG [5].

Interestingly, we found a high prevalence of AKI stage 1 (82.9% of AKI patients) and that even this slight increase in creatinine was associated with an increase in long-term mortality (HR 1.4 (95% CI: 1.0 to 2.1)).

Suggested short- and long-term pathophysiologic mechanisms between AKI and cardiovascular events include fluid retention leading to unstable heart function and inflammation leading to apoptosis and fibrosis at the cardiac level [20,21]. It is known that chronic kidney disease increases the risk of adverse cardiac events [22]. However, whether the effect of AKI is mediated by the development of chronic kidney disease is still not evident and ideally requires prospective and regular measurement of creatinine after discharge. Clinical studies of adverse cardiac events after AKI is sparse and no studies have used time-to-event analysis to examine the prognostic impact of AKI on the risk of myocardial infarction in cardiac surgical patients. The aforementioned study by Tsai *et al*. found a higher risk of major adverse cardiac events after one year among AKI patients (40% (54 of 135)) compared with non-AKI patients (15% (18 of 121)) [7]. Similarly, they found a higher risk of stroke among AKI patients. Studies of patients undergoing coronary angiography and percutaneous coronary intervention have also found a substantially higher risk of myocardial infarction during long-term follow-up [23-25]. This indicates that the long-term prognostic impact of AKI appears consistent, although the prevalence of AKI differs according to population under study.

### Strengths and limitations

The strengths of our study include a well-defined study population with uniform access to health care which minimizes selection bias. Our study population consisted of solely elective surgical patients, thereby making a homogenous cohort of patients. It is, therefore, reasonable to assume that the patients’ pre-conditions and immediate risks of AKI were more alike than if the study population also included acute patients. We had complete pre-operative plasma creatinine measurements as an estimate of baseline kidney function and due to the elective properties of the study population, this measured baseline creatinine was reliable as a good estimate of the patients’ real baseline level. Furthermore, we had detailed pre-, peri- and post-operative data.

We were not able to include all patients undergoing surgery in the study period, but patient screening and recruitment was done by a project nurse whose working schedule was independent of which patients were on the surgery schedule for the day, hence minimizing selection bias. Furthermore, the urine output criteria were not applied to determine AKI status.

We defined the outcomes myocardial infarction and stroke by ICD-10 codes. The positive predictive value was above 92% for myocardial infarction and 80% for stroke [26,27]. Overall, these indicate that we encountered few false positive outcomes; hence risk of information bias was limited. However, if present, this misclassification would presumably be non-differential, and bias the association towards unity.

Due to lack of registration of an exact event date, a patient receives the code of diagnosis at hospital discharge. For the purpose of a causal interpretation between AKI and MI/stroke, we only encountered the myocardial infarction/stroke cases if the outcome of interest occurred after discharge from the index admission for surgery. Meaning, we excluded all patients with an outcome during the index admission for surgery. In this manner we assured that the outcome occurred after the AKI, which is required for a causal interpretation.

For every patient follow-up began on the fifth post-operative day. Due to the definition of the outcomes for myocardial infarction/stroke (only encountering outcomes at a new hospitalization after the index admission for surgery) an immortal person-time bias was introduced, where the object of study was not able to experience an outcome [28]. Particularly, this may be the case for patients with long hospitalizations. Our estimates may, therefore, be underestimated. However, the median length of hospital stay for AKI patients was only seven days and five days for non-AKI patients.

When adjusting for propensity scores and propensity score matching we were able to control for the potential confounding caused by the covariates included in the propensity score, that is, patient-related factors, life-style factors, disease history and surgery-related factors. Still, we cannot exclude the possibility of residual or unmeasured confounding. However, we do believe that we have addressed the most important confounders in our analyses.

Finally, our study population was of limited size; thus, some of our estimates are accompanied by broad confidence intervals.

### Clinical perspectives

This study demonstrates the impact of early post-operative AKI on mortality, specifically in elective patients without pre-operative severe kidney disease. This finding should encourage initiatives towards developing prophylactic strategies for patients who develop even mild reductions in kidney function. However, the risk of myocardial infarction and stroke is still uncertain after this study. Whether the potentially increased risk reflects the effect of AKI or whether AKI acts as a marker of vulnerability remain unclear. The study is most likely generalizable throughout the setting of elective cardiac surgery.

## Conclusion

AKI following elective cardiac surgery was associated with increased five-year mortality, and the risk increased with increasing AKI stage. Although statistically imprecise, AKI may be associated with an increased risk of myocardial infarction, but there was no association with the risk of stroke.

## Key messages

• More than one out of four elective cardiac surgery patients developed AKI within the first five days after cardiac surgery.

• AKI following elective cardiac surgery was associated with increased five-year mortality.

## Abbreviations

AKI: Acute kidney injury; AKIN: Acute kidney injury network; BMI: Body mass index; CABG: Coronary artery bypass grafting; CAG: Coronary angiography; CCI: Charlson comorbidity index; CI: Confidence interval; DNRP: Danish national registry of patients; EuroSCORE: European system for cardiac operative risk evaluation; GFR: Glomerular filtration rate; HR: Hazard ratio; ICD: International classification of disease; PCI: Percutanous coronary intervention; RIFLE: Risk, injury, failure, loss of function, end-stage renal disease.

## Competing interests

The authors declare that they have no competing interests.

## Authors’ contributions

MKH participated in the study design, development and methodology, data analysis and interpretation, and manuscript writing. HG participated in the study design, development and methodology, data analysis and interpretation, and manuscript revision. MMM and VEH participated in the study design, data collection and manuscript revision. JBL contributed to the development and methodology, data analysis and interpretation, and manuscript revision. SPJ and CFC participated in the study design, development and methodology, data analysis and interpretation, and manuscript revision. All authors read and approved the final manuscript.

## Supplementary Material

Additional file 1 **Identification of outcomes and causes of death.** Codes used to identify the studied outcomes and causes of death according to the International Classification of Disease 10^th^ revision. Click here for file

Additional file 2 **Charlson conditions and the corresponding International Classification of Disease (ICD) codes, 10**^**th**^** and 8**^**th**^**revision.** Charlson comorbidities and the codes used to identify the diseases. The codes are according to the International Classification of Disease 8^th^ (1977 to 1993) and 8^th^ (1994-) revision. Click here for file

Additional file 3 **Development in eGFR after elective cardiac surgery.** Box and whiskers plot of the estimated glomerular filtration rate (eGFR) during follow-up. eGFR was calculated by the Modification of Diet in Renal Disease equation by using the available plasma creatinine measures [29]. For each period the highest plasma creatinine measure was chosen if a patient had more than one measure. Click here for file
