# Supplementary material for: Post-operative acute kidney injury and five-year risk of death, myocardial infarction, and stroke among elective cardiac surgical patients: a cohort study
Source: Crit Care. 2013 Dec 12;17(6):R292. doi: 10.1186/cc13158 (PMC4057271; doi:10.1186/cc13158)
Supplement: Additional file 1 — Identification of outcomes and causes of death. Codes used to identify the studied outcomes and causes of death according to the International Classification of Disease 10th revision. [file cc13158-S1.pdf]

Additional file 1. Identification of outcomes and causes of death.

**Outcome**	**ICD-10 code**
Myocardial infarction	I21
Stroke	I61, I63, I64
**Cause of death**	
Myocardial infarction	ICD-10: I21
Chronic ischemic heart disease	ICD-10: I25
Heart failure	ICD-10: I50
Other heart diseases	ICD-10: I00-20, I23-24, I26-49, I51-52
Cerebrovascular disease	ICD-10: I60-69
Kidney insufficiency	ICD-10: N17-19
Sepsis	ICD-10: A41
Tumor	ICD-10: C00-95, D46-47
Other	All other codes not included above

Abbreviations: ICD: International Classification of Diseases 10th revision
