# Supplementary material for: Post-operative acute kidney injury and five-year risk of death, myocardial infarction, and stroke among elective cardiac surgical patients: a cohort study
Source: Crit Care. 2013 Dec 12;17(6):R292. doi: 10.1186/cc13158 (PMC4057271; doi:10.1186/cc13158)
Supplement: Additional file 2 — Charlson conditions and the corresponding International Classification of Disease (ICD) codes, 10th and 8threvision. Charlson comorbidities and the codes used to identify the diseases. The codes are according to the International Classification of Disease 8th (1977 to 1993) and 8th (1994-) revision. [file cc13158-S2.pdf]

Additional file 2. Charlson conditions and the corresponding codes after the International Classification of Disease (ICD), 10th and 8th revision.[a]

Charlson conditions	Corresponding ICD-10 codes	Corresponding ICD-8 codes	Weight
Myocardial infarction	I21, I22, I23	410	1
Congestive heart failure	I50, I11.0, I13.0, I13.2	427.09, 427.10, 427.11, 427.19, 428.99	1
Peripheral vascular disease	I70, I71, I72, I73, I74, I77	782.49, 440, 441, 442, 443, 444, 445	1
Cerebrovascular disease	I60-69, G45, G46	430-438	1
Dementia	F00-F03, F05.1, G30	290.09-290.19, 293.09	1
Chronic pulmonary disease	J40-J47, J60-67, J68.4, J70.1, J70.3, J84.1, J92.0, J96.1, J98.2, J98.3	490-493, 515-518	1
Connective tissue disease	M05, M06, M08, M09, M30, M31, M32, M33, M34, M35, M36, D86	712, 716, 734, 446, 135.99	1
Ulcer Disease	K22.1, K25-28	530.91, 530.98, 531-534	1
Mild liver disease	B18, K70.0-K70.3, K70.9, K71, K73, K74, K76.0	571, 573.01, 573.04	1
Diabetes Mellitus	E10.0, E10.1, E10.9 E11.0, E11.1, E11.9	249.00, 249.06, 249.07, 249.09, 250.00, 250.06, 250.07, 250.09	1
Hemiplegia	G81, G82	344	2
Moderate/severe renal disease	I12, I13, N00-N05, N07, N11, N14, N17-N19, Q61	403, 404, 580-583, 584, 590.09, 593.19, 753.10-753.19, 792	2
Diabetes Mellitus with chronic complications	E10.2-E10.8 E11.2-E11.8	249.01-249.05, 249.08, 250.01.250.05, 250.08	2
Any tumor	C00-C75	140-194	2
Leukemia	C91-C95	204, 205, 206, 207	2
Lymphoma	C81-C85, C88, C90, C96	200, 201, 202, 203, 275.59	2
Moderate/severe liver disease	B15.0, B16.0, B16.2, B19.0, K70.4, K72, K76.6, I85	070.00, 070.02, 070.04, 070.06, 070.08, 573.00, 456.00-456.09	3
Metastatic solid tumor	C76-C80	195-198, 199	6
AIDS	B21-B24	079.83	6

[a] Usage of ICD-8 between 1977-1993, usage of ICD-10 since 1994.
