# Supplementary figures and images for: Post-operative acute kidney injury and five-year risk of death, myocardial infarction, and stroke among elective cardiac surgical patients: a cohort study
Source: Crit Care. 2013 Dec 12;17(6):R292. doi: 10.1186/cc13158 (PMC4057271; doi:10.1186/cc13158)

## Slide 1
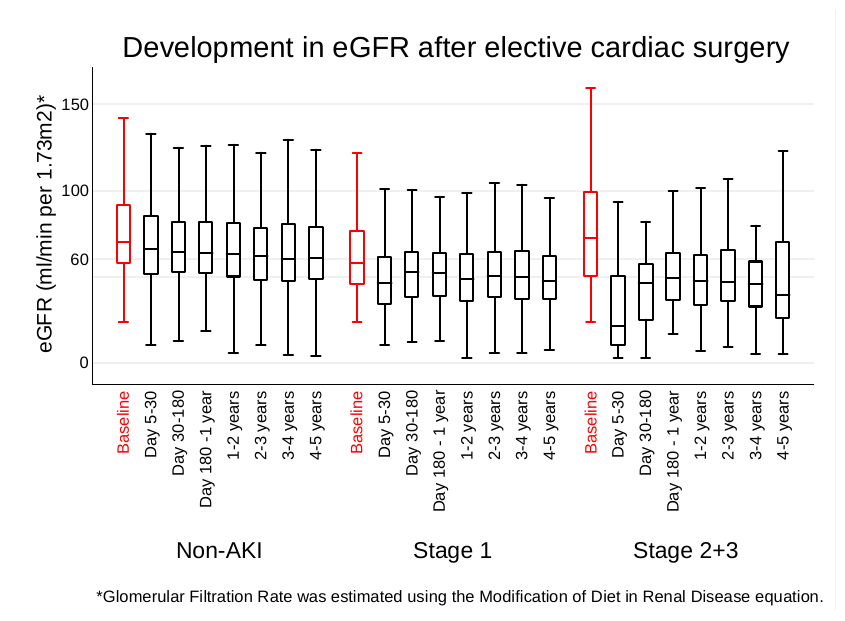

Supplement: Additional file 3 — Development in eGFR after elective cardiac surgery. Box and whiskers plot of the estimated glomerular filtration rate (eGFR) during follow-up. eGFR was calculated by the Modification of Diet in Renal Disease equation by using the available plasma creatinine measures [29]. For each period the highest plasma creatinine measure was chosen if a patient had more than one measure. [file cc13158-S3.pptx]
